# Use of a Sampling Area-Adjusted Adenosine Triphosphate Bioluminescence Assay Based on Digital Image Quantification to Assess the Cleanliness of Hospital Surfaces

**DOI:** 10.3390/ijerph13060576

**Published:** 2016-06-09

**Authors:** Yu-Huai Ho, Lih-Shinn Wang, Hui-Li Jiang, Chih-Hui Chang, Chia-Jung Hsieh, Dan-Chi Chang, Hsin-Yu Tu, Tan-Yun Chiu, Huei-Jen Chao, Chun-Chieh Tseng

**Affiliations:** 1 Division of Infection Diseases, Department of Internal Medicine, Buddhist Tzu Chi General Hospital and Tzu Chi University; Hualien 97004, Taiwan; yuhuai@tzuchi.com.tw (Y.-H.H.); lihshinn_wang@tzuchi.com.tw (L.-S.W.); 2 Unit of Infection Control and Management, Buddhist Tzu Chi General Hospital, Hualien 97004, Taiwan; jhl7557@tzuchi.com.tw; 3 Department and Graduate Institute of Public Health, Tzu Chi University, Hualien 97004, Taiwan; 100313038@gms.tcu.edu.tw (C.-H.C.); cjhsieh@mail.tcu.edu.tw (C.-J.H.); 100313033@gms.tcu.edu.tw (D.-C.C.); 100313039@gms.tcu.edu.tw (H.-Y.T.); 100313008@gms.tcu.edu.tw (T.-Y.C.); 4 Department of Laboratory Medicine, Buddhist Tzu Chi General Hospital, Hualien 97004, Taiwan; jhj03070@tzuchi.com.tw

**Keywords:** adenosine triphosphate, relative light units, infection control, cleanliness

## Abstract

Contaminated surfaces play an important role in the transmission of pathogens. We sought to establish a criterion that could indicate “cleanliness” using a sampling area–adjusted adenosine triphosphate (ATP) assay. In the first phase of the study, target surfaces were selected for swab sampling before and after daily cleaning; then, an aerobic colony count (ACC) plate assay of bacteria and antibiotic-resistant bacteria was conducted. ATP swabs were also tested, and the ATP readings were reported as relative light units (RLUs). The results of the ACC and ATP assays were adjusted according to the sampling area. During the second phase of the study, a new cleaning process employing sodium dichloroisocyanurate (NaDCC) was implemented for comparison. Using the criterion of 2.5 colony-forming units (CFU)/cm^2^, 45% of the sampled sites were successfully cleaned during phase one of the study. During phase two, the pass rates of the surface samples (64%) were significantly improved, except under stringent (5 RLU/cm^2^) and lax (500 RLU) ATP criteria. Using receiver-operating characteristic curve analysis, the best cut-off point for an area-adjusted ATP level was 7.34 RLU/cm^2^, which corresponded to culture-assay levels of <2.5 CFU/cm^2^. An area adjustment of the ATP assay improved the degree of correlation with the ACC-assay results from weak to moderate.

## 1. Introduction

Contaminated surfaces in hospital rooms play an important role in the transmission of pathogens. Routine housekeeping practices are important to reduce the risk of transmission, but several studies have found that these practices are suboptimal and difficult to verify because there are no measurable standards [1,2].

Several methods have been used to assess environmental cleanliness; one of these methods is the aerobic colony count (ACC) assay, which reveals the amounts of culturable bacteria present on the surface of interest. The original quantitative ACC-based standard for defining the surfaces in a ward environment as clean was less than 5 colony-forming units (CFU)/cm^2^, but this value has been reduced to 2.5 CFU/cm^2^ [3,4,5]. Although ACC assay results remain the primary values indicating the “cleanliness” of the surface, completing the ACC assay requires several days. To better quantify the microbial load for exposure assessment, both culture and non-culture methods should play equally important roles.

Currently, the non-culture adenosine triphosphate (ATP) bioluminescence assay is extensively used to evaluate cleanliness because readings can be obtained on site. Because of its presence in living organisms, ATP was first used as an indicator of cleanliness in the food industry [6]. Subsequently, ATP measurements have been employed to assess hospital cleanliness using different benchmark values expressed in relative light units (RLUs). Surface cleanliness has been defined based on thresholds ranging from 100 to 500 RLU [2]. However, the surface area varied widely [7], and the thresholds may have been affected by the use of different ATP-measurement devices and sampling methods [1,4,8]. Notably, except in a few studies [7,9], the threshold of the ATP-assay results used to indicate cleanliness was not calibrated according to the sampling area. The RLU value that is obtained using the ATP assay is a relative unit that has not been quantified against known standards [10]. Similar to culture level values, the measured ATP levels should be proportional to the sampling area, and if the ATP readings are obtained without calibration of the sampling area, the values will be difficult to interpret and the correlation with ACC assay–based values will decrease. Therefore, determining the sampling area may be important, particularly when swab sampling is performed on surfaces that are small or have a non-uniform shape. However, no studies have evaluated the effect of sampling area on the obtained RLU values due to the lack of an appropriate method for quantifying the sampling area.

Sodium hypochlorite (NaOCl), which is household bleach, has been widely used for environmental cleaning and has been demonstrated to effectively reduce the level of contamination on surfaces. However, the disadvantage of NaOCl is related to its toxicity to humans, such as irritating the skin, conjunctiva, or respiratory system. At present, there is one possible alternative to NaOCl, sodium dichloroisocyanurate (NaDCC), which has been approved by the World Health Organization and the United States Environmental Protection Agency for the treatment of drinking water [11]. Compared to NaOCl, NaDCC offers advantages in terms of cost, safety, stability and convenience [11]. A number of studies have also found that NaDCC yielded superior results compared to NaOCl for inactivating several microbial species [12,13]. Thus, whether NaDCC is appropriate for application in hospital cleaning is worthy of study.

The aim of this investigation was to conduct a two-phase study to evaluate the cleanliness of hospital surfaces after two different cleaning procedures were performed. In phase one of the study, the efficiency of the standard daily procedures of cleaning surfaces using bleach was assessed. In phase two of the study, bleach was replaced by NaDCC. Therefore, we hypothesized that employing NaDCC and surveillance would result in improved cleaning practices. The assessment methods included both an ACC assay and a sampling area–adjusted ATP assay based on digital image quantification. In addition to the ACC and ATP assays, specific culture assays to detect carbapenem-resistant *Acinetobacter baumannii* (CRAB), methicillin-resistant *Staphylococcus aureus* (MRSA) and vancomycin-resistant *Enterococcus* spp. (VRE) were also conducted to determine the efficiency of cleaning during the two study periods. Finally, the results obtained during the two study periods were merged and analyzed to identify a criterion that could indicate “cleanliness” in hospital environments.

## 2. Materials and Methods

### 2.1. Phase One

#### 2.1.1. Hospital Cleaning Methods

For this study, we obtained permission from the department of infection control and management of Tzu Chi Hospital. The first phase of the study was designed to assess the performance of the usual daily cleaning procedures using ACC and ATP assays in addition to culture assays specific for CRAB, MRSA, and VRE. A private cleaning company used microfiber cloths for the routine cleaning and applied 0.06% sodium hypochlorite (NaOCl) as a detergent disinfectant. In this phase of the study, the housekeepers were not informed that their cleaning practices were being monitored.

#### 2.1.2. Sampling Sites and Determining the Sampling Areas

**Figure 1:**
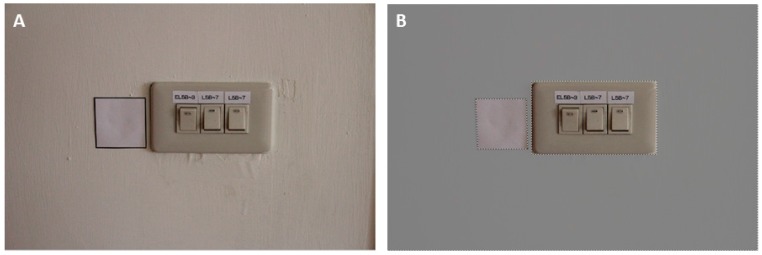
Raw (**A**) and processed photographs (**B**) that were used to determine the surface area of a light switch. The number of pixels in the reference paper (5 × 5 cm) and the surface of interest were calculated using the processed photographs. The equation used to calculate the surface area is described in the Methods Section.

During the first phase of the study, the following 11 target surfaces in patient rooms were selected for swab sampling before and after daily cleaning: bedside rails, bedside tables, chairs, doorknobs, drawer handles, emergency buttons, light switches, hand sanitizer pump, toilet flush handles, toilet safety rails and wardrobe handles. In total, 121 sampling sites in the rooms of 11 randomly selected patients were examined. Because the colony counts and the observed RLU values are proportional to the sampling area, the obtained colony-count and RLU values were all adjusted according to the sampling area. For sampling sites with a spherical or cylindrical shape, such as doorknobs or toilet safety rails, the radius was measured and used in a mathematical formula to calculate the surface area. The areas of flat sampling sites were quantified by counting the number of pixels in a digital image of the target, similarly to a method that has been used to measure leaf areas in ecological and environmental studies [14,15] (Figure 1). Briefly, we placed a 5 × 5 cm white reference paper next to the target surface and captured an image using a common digital camera. To capture the image, the target surface and the camera were aligned vertically with the focus aimed at the center of the target. We obtained images including both the reference paper and the surface of interest and then determined the number of pixels in each target using Photoshop software (Adobe Systems Incorporated, San Jose, CA, USA). To count the pixels in a digital image, an image histogram was generated using the “Histogram” function. Then, the area of the surface of interest was obtained using the following equation:

$$\frac{A_{\text{surface}}}{A_{\text{reference paper}}}=\frac{N_{\text{surface}}}{N_{\text{reference paper}}}$$

where A_surface_ and A_reference paper_ are the areas of the target surface and the reference paper (25 cm^2^), respectively, and N_surface_ and N_reference paper_ are the number of pixels in the images of the target surface and the reference paper, respectively, which were counted in the same photograph using Photoshop.

#### 2.1.3. ATP Bioluminescence and Microbiological Assessment

The ATP bioluminescence kit was purchased from 3M (3M Clean-Trace ATP System; 3M Co., St. Paul, MN, USA). Before and after the daily cleaning was performed, samples were collected using ATP swabs from fully dried surfaces of areas immediately adjacent to the areas sampled for the culture assays. After sampling, the ATP swabs were placed in ATP bioluminescence reaction tubes and agitated to allow the reaction to occur. Then, the reaction tubes were inserted into a luminometer, and ATP readings were obtained and expressed in RLU. We then converted the RLU values into RLU/cm^2^ by adjusting for the surface area.

The ACC assay was used as an indicator of the general bacterial load. The samples for the total aerobic colony counts were collected using sterile moistened swabs that were then placed in 1 mL of phosphate-buffered saline and immediately transferred to the laboratory for culture. Aliquots (0.1 mL) of the culture samples were diluted 10- to 100-fold, plated on tryptic soy agar (TSA) plates [16] in duplicate and then incubated for 48 h at 37 °C. Finally, the total aerobic colony counts of the target surfaces were calculated based on the dilution ratio, and the counts were adjusted according to the surface areas to obtain the results in CFU/cm^2^.

In addition to the effect of cleaning on the microbial load, which was assessed using the ACC and ATP assays, the presence of CRAB, MRSA and VRE on the target surfaces before and after the daily cleaning was also evaluated. To rapidly identify CRAB, MRSA, and VRE strains on the surfaces, the chromogenic agars CHROMagar Acinetobacter, CHROMagar MRSA, and CHROMagar VRE (CHROMagar Microbiology, Paris, France) were used. The *A. baumannii* strains that were recovered on the selective chromogenic agar were identified using a Vitek system (bioMérieux, Marcy-l’Etoile, France). The resistance of *A. baumannii* to carbapenem was confirmed using the broth-dilution method in accordance with the 2012 Clinical and Laboratory Standards Institute guidelines. To specifically detect MRSA, coagulase testing was performed to exclude species that were coagulase-negative or that had a Gram-staining property that was not consistent with MRSA [17]. In addition, colonies that appeared mauve-positive on CHROMagar VRE were evaluated using conventional laboratory tests to verify their identity as VRE [18].

### 2.2. Phase Two

In the second phase of our study, we implemented a new cleaning process in which sodium dichloroisocyanurate (NaDCC) tablets (Medentech, Wexford, Ireland) were used as an alternative disinfectant. We dissolved one NaDCC tablet in 1 L of water, thus creating a concentration of available chlorine equal to that found in 0.05% NaOCl. The introduction of the NaDCC process was led by an infection-control nurse. Due to the NaDCC introduction, the housekeeping staff may have known that that the target surfaces would be monitored. However, the rooms chosen for monitoring were randomly selected and were not necessarily the same rooms evaluated in phase one of the study. The ACC and ATP surveillance results were not disclosed to any of the medical and housekeeping staff. During this second phase, microfiber cloths were still used for the cleaning, and the sampling and analytical methods were the same as those used during the first phase of the study.

### 2.3. Test Standards and Pass Rates

The two most commonly used thresholds for cleanliness evaluations using the ATP assay are 250 RLU [1,3,5,19,20,21] and 500 RLU [4,6,21], but the surface areas widely vary. If the thresholds had been adjusted according to the sampling area (typically 100 cm^2^), the thresholds proposed in two previous reports might be 5 RLU/cm^2^ [22] or 10 RLU/cm^2^ [23]. When the ACC assay was used, 2.5 CFU/cm^2^ was the standard threshold because it has been recommended as a criterion for defining surfaces as clean [3,5,7,20,22]. In the case of detecting CRAB, MRSA and VRE strains, the absence of these three antibiotic-resistant species was defined as clean [22]. Therefore, the pass rates can be calculated as the percentage of ATP or culture samples in which the obtained values were less than the recommended thresholds.

### 2.4. Statistical Analysis

Because the sample sizes in both phase one (*n* = 726) and phase two (*n* = 714) were larger than 50, we used the Kolmogorov-Smirnov test to determine whether the sample data were normally distributed. After statistical analysis, non-parametric tests were used for the data analysis because the probability associated with the Kolmogorov-Smirnov test of normality is <0.05. The differences between the RLU/cm^2^ and CFU/cm^2^ values before and after daily cleaning were compared using the Wilcoxon signed-rank test to determine whether they were significant (*p* < 0.05). We also used Spearman’s rank correlation (r = Spearman’s rho) to assess the correlation between the culture levels and ATP levels before and after adjusting for the sampling area. In addition, the difference between the pass rates during the two phases of the study was determined using a chi-squared test. Receiver-operating characteristic (ROC) curves, with the ACC assay as the standard, were generated to assess how well each ATP assay (with and without adjustment for the sampling area) could predict the subsequent culture levels. The results obtained during both phases of the study were included in this analysis.

## 3. Results

For all 11 sampling sites, the mean proportion of surfaces with an ATP level after cleaning that was lower than the level before cleaning was 74.4% (from 45.5% to 95.9%) in phase one of the study (Table 1). A similar proportion of 74.7% (from 54.6% to 100%) was also observed in phase two of the study (Table 1). The ATP levels on bedside tables, chairs and light switches after cleaning were significantly lower than the levels before cleaning during the first phase of the study. After the introduction of the new cleaning process, a significant reduction in the ATP levels after cleaning was observed on bedside tables, doorknobs and emergency buttons.

The proportion of surfaces with a culture-based level after cleaning that was lower than the pre-cleaning level was 70.2% (from 36.4% to 90.9%) during phase one of the study (Table 2). A slight increase in this proportion to 76.0% (from 54.6% to 100%) for all surfaces was observed during phase two of the study (Table 2). During the first phase, the sample sites on which the culture-based levels obtained after cleaning were significantly lower than those obtained before cleaning were the bedside rails, bedside tables, chairs, doorknobs, and light switches. After the introduction of the new cleaning process, the culture-based levels on the bedside tables, doorknobs, drawer handles, and hand sanitizer pump were significantly lower than the pre-cleaning levels.

Table 3 shows the culture-based values of CRAB, VRE and MRSA on the pre-cleaning and post-cleaning surfaces during the two study periods. Not all of the evaluated surfaces harbored all three of these antibiotic-resistant species. Therefore, the number of samples on which the respective bacterial species were detected on the pre-cleaning or post-cleaning surfaces is shown. During the two study periods, MRSA was the predominant species recovered from 93 (38.4%) surfaces. Although CRAB was not the most commonly found species in this study, the concentration of CRAB was approximately 5.6 to 103 times higher than those of VRE and MRSA before cleaning. Among the surfaces found to be colonized by these antibiotic-resistant bacteria (*n* = 120), 43 (35.8%) were near a patient who was infected with CRAB, VRE or MRSA. During both of the study periods, the post-cleaning concentrations of all three of these antibiotic-resistant bacteria were significantly lower than the pre-cleaning concentrations.

Table 4 shows how the pass rates of all of the sampled surfaces varied according to different criteria. Using the criterion of 2.5 CFU/cm^2^, 64% of the sampled sites were successfully cleaned after the introduction of the new cleaning process. When the ATP assay was used, the cleanliness pass rate based on the criterion of 5 RLU/cm^2^ was improved from 39% to 52% after the introduction of the new cleaning process, but the level of change was only at the edge of significance (*p* = 0.052). The cleanliness determined using the other two criteria (10 RLU/cm^2^ and 250 RLU) also agreed with that for the criterion of 2.5 CFU/cm^2^, and the new cleaning process significantly improved the pass rates. However, the pass rates before (71%) and after (81%) introducing the new cleaning process did not change significantly for a lenient criterion of 500 RLU.

By qualitative standards, no antibiotic-resistant pathogen should be present on the surfaces that we tested. Table 5 shows that there was no significant difference in the pass rates for all three of the antibiotic-resistant pathogens before and after the new cleaning process was introduced. For CRAB and VRE, there was a high pass rate of 88%–91% during phase one of the study. However, the pass rate for MRSA was unsatisfactory both before and after the new cleaning process was introduced (54% *vs.* 52%; *p* = 0.703).

**Figure 2:**
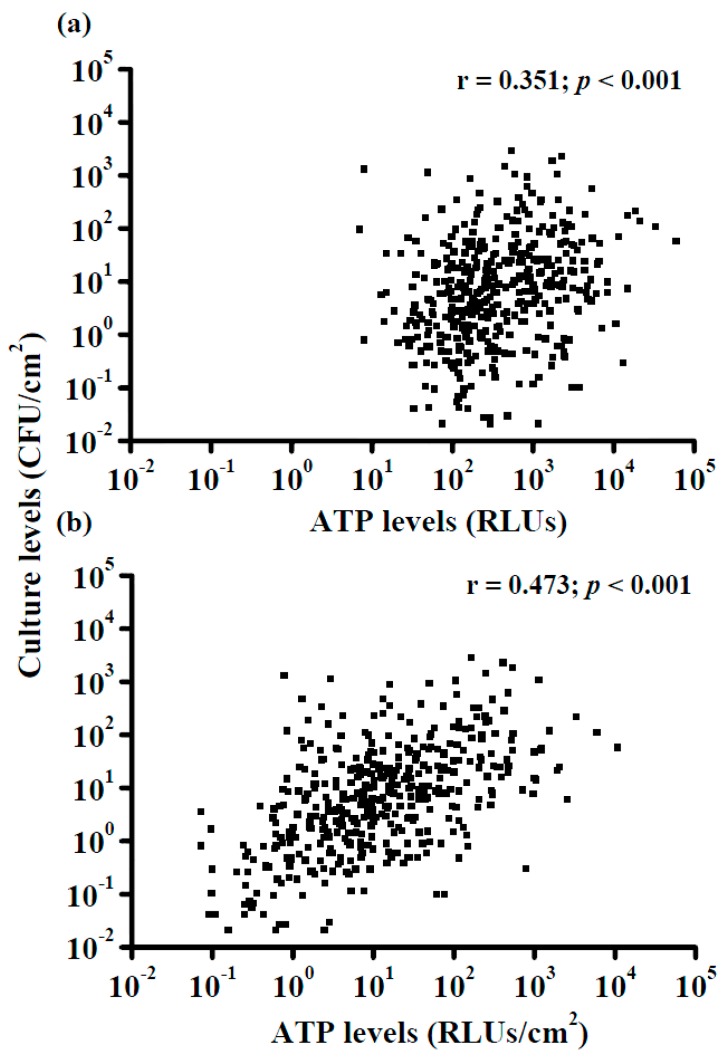
Correlation between the culture-based levels and either (**a**) the non-adjusted ATP-based (RLU) levels or (**b**) the adjusted ATP-based levels (RLU/cm^2^) obtained from 11 sampling sites in 22 patient rooms.

Figure 2a shows that when the ATP levels were not adjusted according to the sampling area, the correlation with the culture-based levels was significant (*p* < 0.001) but weak (*r* = 0.35). However, after the ATP levels were adjusted for the sampling area (Figure 2b), the correlation with the culture-based levels was significant (*p* < 0.001), and the degree of correlation was improved from weak to moderate (*r* = 0.47).

**Figure 3:**
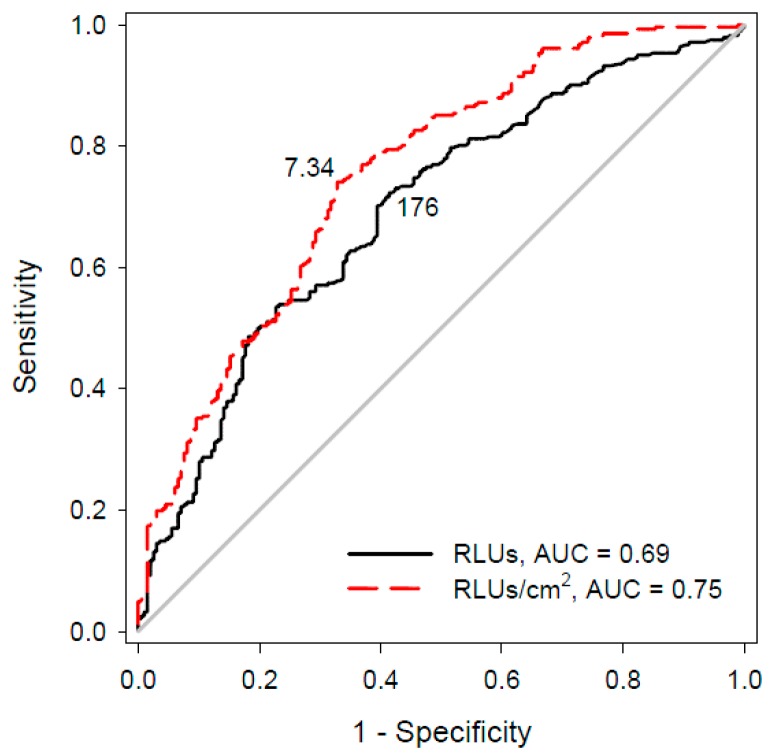
Receiver-operating characteristic (ROC) curves of the criteria based on the non-adjusted ATP-based values (RLU; solid black line) and adjusted ATP-based levels (RLU/cm^2^; dashed red line) *versus* the microbial growth-based values. Culture-based levels of >2.5 CFU/cm^2^ were regarded as culture positive. The best cut-off points demonstrated by both curves were determined using the Youden index. AUC refers to the area under the curve.

The ROC curve analysis (Figure 3) showed that all of the possible criteria corresponding to the ATP values (with or without a sampling-area adjustment) could predict the subsequent culture-based positive (>2.5 CFU/cm^2^) results. ATP values that were adjusted according to the sampling area appeared to be a better predictor than the non-adjusted ATP values (area under the curve (AUC) = 0.75 > 0.69; *p* = 0.01). An AUC value higher than 0.70 indicated that the area-adjusted ATP values provided acceptable discrimination of microbial growth. The best cut-off point determined using the Youden index was 7.34 RLU/cm^2^ for the area-adjusted ATP level (sensitivity: 74%; specificity: 67%) and 176 RLU for the non-adjusted ATP level (sensitivity: 72%; specificity: 58%); these values correspond to culture-based levels of <2.5 CFU/cm^2^. The sensitivity level of 74% determined using ROC curve analysis indicates that 74% of the ATP-assayed samples with values that were higher than 7.34 RLU/cm^2^ yielded a culture-based positive result (>2.5 CFU/cm^2^). Likewise, the specificity level of 67% indicates that 67% of the ATP-assayed samples with values that were lower than 7.34 RLU/cm^2^ yielded a culture-based negative result (<2.5 CFU/cm^2^).

## 4. Discussion

The difference between the culture-based levels before and after cleaning during the second phase of the study was significantly lower than that observed during the first phase of the study for all of the surfaces (*p* < 0.001). This result may be related to the use of NaDCC. However, these differences were not observed in the ATP-based levels (*p* = 0.06), and previous studies have indicated that the ATP-based or culture-based levels observed after cleaning may occasionally be higher than those observed before cleaning [1,4,10]. With the exception of the possible variability in the ATP assay [10], it is likely that dirt and/or microorganisms were redistributed rather than removed by cleaning [24]. In addition, only a small number of surfaces were significantly cleaner after cleaning during both study periods. These results indicate that both cleaning processes may not be optimal. For example, the cleaning performance could not significantly improve if the monitoring results were not provided to the housekeepers so that they could immediately correct their cleaning practices. Feedback has been demonstrated to be important in reducing MRSA and VRE contamination [25,26].

In fact, it is more important that the ATP- or culture-based levels reach a safe level after cleaning than it is that the levels decrease by a certain amount. Many authors have proposed using ACC values of 2.5 CFU/cm^2^ as a criterion for defining surfaces as clean [3,5,7,20,22]. Our results agreed with those of previous studies in that there was a low degree of correlation between the culture-based results and the ATP levels [1,3,5]. However, when the results were adjusted for the sampling area, the correlation between the ATP- and culture-based levels improved. The ACC assay only measures aerobic microorganisms, whereas the ATP assay detects not only microorganisms but also many forms of organic material. Nevertheless, the measured ATP levels should be proportional to the sampling area, similar to the culture-based levels. Therefore, it would be beneficial to adjust for the sampling area before using ATP results.

The ATP levels were adjusted according to the sampling area in only a few studies in which sampling was conducted on large surfaces of 100 cm^2^ [9,22,23]. Non-adjusted ATP levels might diminish the ability to compare the results of different studies. For example, according to the most commonly used ATP benchmarks of 250 and 500 RLU, our cleanliness pass rates after the introduction of the new cleaning process (72% and 81%, respectively) were lower than those of Moore *et al.* in a study conducted on Intensive Care Unit (ICU) surfaces (90% and 95%, respectively) [21]. This result may be related to ICU cleanliness requirements being more stringent than the requirements for common wards, but an objective comparison cannot be made because the sampling areas were different. Additionally, it seems that Sherlock *et al.* used the less stringent benchmark of 500 RLU compared to the 100 RLU benchmark used by Mulvey *et al.* However, after adjusting for the sampling area, the benchmark used by Sherlock *et al.* (5 RLU/cm^2^) was actually more stringent than that used by Mulvey *et al.* (10 RLU/cm^2^).

Our study applied the commonly used criteria for the ACC and ATP assays to survey the pass rate of all of the sites after the intervention. The combination of NaDCC and monitoring may be effective; the surfaces evaluated using the culture-based criterion of 2.5 CFU/cm^2^ were significantly cleaner after the intervention than before the introduction of the new cleaning process. This result was also found using the ATP-assay criteria of 10 RLU/cm^2^ and 250 RLU. The criterion of 5 RLU/cm^2^ was more stringent than our best cut-off point of 7.3 RLU/cm^2^, as indicated in Figure 3; therefore, the pass rate in this study may not have been significantly changed before and after the introduction of the new cleaning process. Moreover, fewer sites would fail if the more lenient criterion of 500 RLU were used. False-negative test results in studies with laxer criteria could increase the infection risk from insufficiently cleaned surfaces [10].

Because the pass rates for CRAB (91%) and VRE (88%) on all of the sites were relatively high, it is difficult to discuss the contribution of the new cleaning process to the removal of these two pathogenic species. However, the results of our study agreed with those of previous studies showing that MRSA was not necessarily removed by cleaning even when the measured ATP levels were lower than the recommended criterion [1,5]. Evaluating healthcare surface cleanliness based on the ATP tool has previously been shown to be ineffective for determining the presence of antibiotic-resistant species [1,5] due to very low levels of these pathogens on healthcare surfaces. Because the median culture values of MRSA in our study were very low (Table 3), it would be difficult to eradicate MRSA from the surface if the cleaning were not comprehensive. In addition, *S. aureus* may have a better tolerance for NaOCl than other bacterial species [27]. Inactivation of *S. aureus* or MRSA in cloth may require a higher concentration of NaOCl or NaDCC than needed for inactivation of other bacterial species. Furthermore, microfiber cloths are not compatible with bleach, possibly explaining the inability to kill MRSA. Interestingly, the pass rates for MRSA on large surfaces, such as bedside rails, bedside tables, and chairs, in phase one of the study were considerably better than those obtained after the introduction of the new cleaning process (*p* < 0.001). However, the cleanliness of some small sites, such as the hand sanitizer pump, doorknobs, emergency buttons and toilet flush handles, was significantly improved after the new cleaning process was introduced (*p* < 0.001). This result may have occurred because the housekeepers were notified of the sites that would be monitored and therefore tended to neglect the sites that they had originally cleaned.

Although the identification of a clean cut-off is somewhat arbitrary, it could be helpful to ensure the effectiveness of cleaning. An area-adjusted ATP-assay result of 7.34 RLU/cm^2^ might be an appropriate criterion when using the 3M system for multiple samples. ROC curve analysis indicated that this cut-off point was similar to that found by Smith *et al.* (8 RLU/cm^2^) using the same ATP-measurement instrument [7]. However, ATP levels below our recommended criterion do not necessarily indicate the absence of specific pathogens. Using this criterion, 5%, 25% and 6% of the sites would contain CRAB, MRSA and VRE, respectively. Without adjustment for the sampling area, the cut-off point of 176 RLU observed using ROC curve analysis in this study was different from the value of 100 RLU obtained using a Hygiena system [5]. However, this benchmark would be closer to that of our study after adjustment for the area (10 RLU/cm^2^). Compared with the results of previous studies in which ROC analysis was conducted, our analysis showed a higher sensitivity (72%–74% *vs.* 57%–58%) level, whereas the specificity (58%–67%) level was in the range of those of previous studies (57%–77%) [5,7]. These differences may be related to variations in the background cleanliness. For practical applications, it is necessary to evaluate whether achieving these recommended benchmarks through cleaning is associated with a reduced infection rate. In addition, although the criterion may be affected by the use of different ATP-measurement instruments with different levels of variability, the sampling-area adjustment is still necessary before interpreting these results.

Our study has some limitations. First, alerting the housekeepers could result in the Hawthorne effect. Because the housekeepers may have known that a study was in progress, they could have improved their performance only when they were observed. However, the results of our study were similar to those of previous studies in which the Hawthorne effect could have occurred [1,26]. The improvement in the cleanliness pass rate was continuously sustained throughout phase two of the study, despite our continued surveillance. Second, although our sample size was larger than that of the only two studies that also utilized ROC analysis [5,7], more samples may be needed to determine a definitional boundary for the ATP assay. Finally, although we evaluated a moderately large number of surfaces, we sampled only 22 rooms; therefore, the findings may not apply to all institutions. The cleanliness of surfaces in healthcare cannot be confirmed as a measure of the safety of the surface or of the risk that surface pathogens may transfer to patients.

## 5. Conclusions

Because the ATP assay is not ideal for evaluating cleanliness, we suggest that the impact of the variability of ATP-measurement devices could be reduced by the parallel use of different methods and by the use of a standard sampling strategy. Therefore, a reliable quantification method for determining the sampling area is important for the use of both ACC and ATP assays. Our study used an ATP bioluminescence assay to monitor the performance of cleaning practices and the impact of the new cleaning process. We recommend that the ATP-assay results be adjusted for the sampling area. The method used in this study to determine the areas is easy to perform and has been verified in environmental studies. Therefore, we can objectively measure the surface area using a digital image, particularly when sampling is performed on sites that are small or non-uniform in shape.

## Figures and Tables

**Table 1:** Median RLU values (RLU/cm^2^) before and after daily cleaning during the two study periods and *p*-values determined using the Wilcoxon signed-rank test (*N* = 480).

Surface	Before Intervention			After Intervention		
	Pre-Clean (Range)	Post-Clean (Range)	*p*	Pre-Clean (Range)	Post-Clean (Range)	*p*
bedside rails	17.7 (3.9–144.7)	4.4 (0.6–75.4)	0.06	24.5 (7.2–298.1)	3.3 (0.7–95.0)	0.11
bedside tables	1.9 (0.2–22.6)	0.3 (0.1–3.7)	0.02	1.8 (0.6–7.7)	0.3 (0.2–1.0)	0.00
chairs	10.2 (1.6–259.8)	9.1 (0.2–19.6)	0.04	6.0 (1.2–59.1)	5.3 (0.3–287.4)	0.53
doorknobs	325.2 (31.3–33130)	41.1 (8.0–10698.2)	0.53	36.1 (11.8–1202.9)	12.1 (2.5–160.4)	0.01
drawer handles	49.6 (15.2–260.1)	17.7 (2.4–102.7)	0.05	16.5 (8.5–90.3)	5.2 (2.2–789.6)	0.05
emergency buttons	15.9 (3.4–200.1)	14.2 (2.2–50.2)	0.14	13.7 (2.6–116.5)	4.8 (0.8–21.7)	0.03
light switches	10.7 (2.4–207.3)	5.0 (3.4–16.6)	0.02	5.0 (1.6–114.2)	2.4 (0.4–149.7)	0.09
hand sanitizer pump	0.8 (0.3–6.9)	0.9 (0.3–7.0)	0.86	0.7 (0.3–9.8)	0.1 (0–2.2)	0.26
toilet flush handles	205.0 (9.0–1049.7)	75.2 (27.2–1532.6)	0.21	23.6 (8.5–249.7)	10.8 (2.9–1134.0)	0.29
toilet safety rails	8.5 (0.6–65.4)	5.3 (0.5–17.7)	0.09	1.5 (0.4–162.3)	5.3 (0.2–114.6)	0.93
wardrobe handles	288.6 (16.0–992.0)	80.0 (15.4–2564.0)	0.21	205.5 (4.6–1920.3)	164.6 (012.6–2019.7)	0.86

**Table 2:** Median ACC values (CFU/cm^2^) before and after daily cleaning during the two study periods and *p*-values determined using the Wilcoxon signed-rank test (*N* = 960).

Surface	Before Intervention			After Intervention		
	Pre-Clean (Range)	Post-Clean (Range)	*p*	Pre-Clean (Range)	Post-Clean (Range)	*p*
bedside rails	15.0 (0.4–587.9)	0.7 (0.0–19.3)	0.00	15.9 (0.0–107.5)	3.9 (0.2–3434.2)	0.09
bedside tables	1.7 (0.0–88.3)	0.1 (0.0–7.1)	0.00	6.2 (0.0–248.1)	0.2 (0.0–2.3)	0.00
chairs	34.7 (1.8–330.0)	0.6 (0.0–49.4)	0.00	3.8 (0.0–450.0)	0.9 (0.0–179.4)	0.08
doorknobs	50.9 (0.0–1198.2)	14.3 (0.0–141.1)	0.04	28.6 (0.0–23026.8)	1.8 (0.0–312.5)	0.00
drawer handles	28.4 (0.0–958.2)	1.2 (0.0–2274.0)	0.43	15.8 (0.0–3486.6)	0.9 (0.0–23.9)	0.00
emergency buttons	14.6 (0.0–1761.0)	4.4 (0.0–58.5)	0.12	2.4 (0.0–37.1)	0.0 (0.0–13268.3)	0.09
light switches	10.0 (0.0–34.1)	2.9 (0.0–55.2)	0.02	4.9 (0.0–259.5)	0.8 (0.0–78.6)	0.18
hand sanitizer pump	0.8 (0.0–6.7)	0.3 (0.0–29.3)	0.46	0.5 (0.0–41.6)	0.0 (0.0–17.0)	0.04
toilet flush handles	51.9 (0.0–690.7)	23.9 (0.0–561.4)	0.09	2.1 (0.0–17381.1)	2.8 (0.0–13.2.2)	0.07
toilet safety rails	3.8 (0.0–17.9)	0.8 (0.0–112.7)	0.60	12.8 (0.0–1187.2)	5.9 (0.0–4738.2)	0.59
wardrobe handles	32.3 (0.0–584.6)	9.2 (0.0–3750.8)	0.15	18.5 (0.0–2200.0)	9.2 (0.0–2935.4)	0.81

**Table 3:** Median culture values (CFU/cm^2^) of CRAB, VRE and MRSA before and after daily cleaning during the two study periods.

Species	Before Intervention			Species	After Intervention		
	Pre-Clean (Range)	Post-Clean (Range)	*p*		Pre-Clean (Range)	Post-Clean (Range)	*p*
CRAB (*n* = 2)	101.7 (0.0–201.8)	N.D.	< 0.01	CRAB (*n* = 10)	8.5 (0.0–16.1)	0.5 (0.0–0.6)	< 0.01
VRE (*n* = 11)	0.98 (0.0–32.1)	0.0 (0.0–0.1)		VRE (*n* = 4)	1.5 (0.0–8.4)	N.D.	
MRSA (*n* = 48)	1.1 (0.0–50.0)	0.0 (0.0–6.2)		MRSA (*n* = 45)	0.9 (0.0–447.3)	0.0 (0.0–5.4)	

*n* = number of samples in which the respective bacterial species were detected on either the pre-cleaning or post-cleaning surfaces; N.D. = not detected; *p*-values determined using the Wilcoxon signed-rank test were used to evaluate the difference in concentration of all three antibiotic-resistant bacteria before and after daily cleaning.

**Table 4:** Pass rate of all sampling sites determined by five different criteria before and after the intervention.

Standard	<2.5 CFU/cm^2^ [3,5,7,20,22]	*p* *	<5 RLU/cm^2^ [22]	*p* *	<10 RLU/cm^2^ [23]	*p* *	<250 RLU [1,3,5,19,20,21]	*p* *	<500 RLU [4,6,21]	*p* *
Before intervention	55/121 (45)	0.004	47/121 (39)	0.052	63/121 (52)	0.017	65/121 (54)	0.003	86/121 (71)	0.082
After intervention	78/121 (64)		63/121 (52)		82/121 (68)		88/121 (73)		96/119 (81)	

* chi-squared test; The number in the brackets indicates the references in which this criterion is used. Data are the proportion (%) of the surface samples tested.

**Table 5:** Pass rate of all sampling sites determined by the absence of CRAB, MRSA and VRE before and after the intervention.

Microbial Species	CRAB	*p* *	MRSA	*p* *	VRE	p *
Before intervention	110/121 (91)	0.669	65/121 (54)	0.703	107/121 (88)	0.711
After intervention	112/121 (93)		63/121 (52)		109/121 (90)	

* chi-squared test; Data are the proportion (%) of the surface samples tested.
